# GraphTar: applying word2vec and graph neural networks to miRNA target prediction

**DOI:** 10.1186/s12859-023-05564-x

**Published:** 2023-11-17

**Authors:** Jan Przybyszewski, Maciej Malawski, Sabina Lichołai

**Affiliations:** 1 Sano Centre for Computational Medicine, Czarnowiejska 36, 30-054 Cracow, Poland; 2 Division of Molecular Biology and Clinical Genetics, Faculty of Medicine, Jagiellonian University Medical College, Skawińska 8, 31-066 Cracow, Poland

**Keywords:** Word2vec, Graph neural networks, Target prediction, RNA interference, Graph classification

## Abstract

**Background:**

MicroRNAs (miRNAs) are short, non-coding RNA molecules that regulate gene expression by binding to specific mRNAs, inhibiting their translation. They play a critical role in regulating various biological processes and are implicated in many diseases, including cardiovascular, oncological, gastrointestinal diseases, and viral infections. Computational methods that can identify potential miRNA–mRNA interactions from raw data use one-dimensional miRNA–mRNA duplex representations and simple sequence encoding techniques, which may limit their performance.

**Results:**

We have developed GraphTar, a new target prediction method that uses a novel graph-based representation to reflect the spatial structure of the miRNA–mRNA duplex. Unlike existing approaches, we use the word2vec method to accurately encode RNA sequence information. In conjunction with the novel encoding method, we use a graph neural network classifier that can accurately predict miRNA–mRNA interactions based on graph representation learning. As part of a comparative study, we evaluate three different node embedding approaches within the GraphTar framework and compare them with other state-of-the-art target prediction methods. The results show that the proposed method achieves similar performance to the best methods in the field and outperforms them on one of the datasets.

**Conclusions:**

In this study, a novel miRNA target prediction approach called GraphTar is introduced. Results show that GraphTar is as effective as existing methods and even outperforms them in some cases, opening new avenues for further research. However, the expansion of available datasets is critical for advancing the field towards real-world applications.

## Background

Discovered in 1993 [1], microRNAs (miRNAs) are a family of short, non-coding RNA molecules that have the ability to influence gene expression. In the RNA interference phenomenon, the miRNA strand binds to an Argonaute protein (AGO) [2]. The resulting miRNA-induced silencing complex (miRISC) can target specific mRNAs, depending on the miRNA nucleotide sequence, and inhibit their translation. As a result, miRNAs can influence the expression of certain genes and take part in the regulation of a number of biological processes in the human organism [3]. Crucially, miRNAs are believed to influence the development of cardiovascular [4], oncological [5], and gastrointestinal diseases [6], as well as viral infections [7]. Because of this ability, the study of miRNAs and their respective targets may prove crucial for the design of novel diagnostic and treatment methods. Experimental validation of miRNA–mRNA target pairs (duplexes) interaction is difficult and costly due to the large number of potential interactions that need to be examined. Computational methods that are able to filter out potential pairs that can be later validated experimentally are therefore of utmost importance to streamline the process of discovering valid miRNA–mRNA targets and increase the efficiency of patient diagnosis and treatment in the healthcare of the future.

Earliest computational methods proposed for target prediction employed expert-based knowledge to classify miRNA–mRNA sequence pairs. For this purpose, metrics inspired by the literature on interaction mechanics were calculated based on the composition of each sequence. The most common metrics include site complementarity [8–10], site conservation [11], and free energy estimation [12–14]. After the calculation, the scores were graded using a rule-based system to determine the binding probability between the pair of RNAs considered.

With an increasing number of experimentally validated miRNA–mRNA pairs and the rise of Machine Learning (ML) in other domains, classical ML algorithms were also introduced into target prediction, in hopes to outperform rule-based systems. Just like the latter, ML methods used calculated metrics as input, but the rules for calculation of the binding probability were learned by the ML classifier directly from the training data, as opposed to being preset. The most popular classifiers used for this task include support vector machines (SVM) [15], boosting methods [12, 16], Bayesian probabilistic models [17], and feedforward neural networks [18, 19].

Although widely used, expert-based methods have two main limitations. First, while pre-engineered features have a documented impact on the miRNA–mRNA interaction, the intrinsic mechanisms behind the binding process are not fully understood. Thus, relying solely on this knowledge may prevent algorithms from achieving optimal performance. Second, the need to calculate interaction metrics based on the sequences adds an often tedious computation step to the procedure, increasing the execution time during inference. To address these limitations, a new family of deep learning (DL) methods has emerged that can process and classify raw RNA data without using any a priori knowledge. After training, these models can extract accurate feature representations of the miRNA–mRNA pair and predict the probability of binding. Despite the limitations that deep learning methods face, such as their reliance on high-quality data, the need for substantial computational resources, interpretability challenges, and the common issue of overfitting, the improving quality of datasets has accelerated their adoption for classification tasks in various fields of computational biology, including protein-protein interaction [20], RNA-disease [21, 22], and miRNA–mRNA interaction, which is the specific domain of focus for this study. Various DL architectures have been used for this purpose, including feedforward, convolutional, and recurrent neural networks. Some methods have also utilized pre-trained autoencoders to improve feature extraction.

One of the first methods, DeepTarget [23], trained separate autoencoders on miRNA and mRNA one-hot encoded sequences in an unsupervised manner. The encoders extracted from the trained models were then used to build a feature representation of each sequence, and the concatenation of the features served as input to a recurrent neural network that acted as a classifier. In DeepMirTar [24], a hybrid approach was proposed where expert-based features and one-hot encoded raw sequence data were processed together by a neural network consisting of a pre-trained stacked denoising autoencoder (SdA) [25] and a fully connected layer on top. Notably, the authors used an unsupervised pre-training strategy for the SdA.

In MiRAW [26], authors used a standard autoencoder, but pre-trained it in an end-to-end manner, unlike the layer-by-layer approach of DeepMirTar. Moreover, the miRAW model used only one-hot encoded sequence data as input without any expert features. The architecture consisted of an encoder extracted after pre-training and a set of fully connected layers as a classifier. One of the most recent methods in this family is miTAR [27]. Gu et al. proposed an architecture that combined recurrent and convolutional layers in a single architecture. The one-hot encoded sequences are concatenated and processed by a set of 1D convolutional layers. Next, a max-pooling operator is applied, and the output is inferred through a bidirectional long short-term memory layer (BiLSTM) [28]. The extracted information is then classified by fully connected layers. The authors hypothesize that convolutional layers can extract compact, spatial features, and this ability is complemented by a recurrent layer that excels in learning long-term sequential features. As a result, the combination of these traits is believed to improve the performance of the model.

The duplex representation used by the aforementioned DL methods is essentially a 1D vector of concatenated duplex sequences. Although it has been shown to provide decent results, we believe that it may not be the best way to represent the miRNA–mRNA interaction. Firstly, DL methods operating on sequences in other domains, e.g. in Natural Language Processing (NLP), have moved away from encoding the input using one-hot encoding towards more sophisticated sequence embedding methods, such as word2vec [29], with great success. This idea has already been applied to the analysis of biological sequences but not in the target prediction domain. In this work, we describe a way to apply word2vec with the hope of improving the predictions of our DL model. Secondly, in reality, miRISC molecules bind directly to the targeted RNAs, creating spatial, graph-like secondary structures. To this end, to improve on existing target prediction methods, we propose a novel DL method that exploits the graph representation of the duplex. The method can classify miRNA–mRNA pairs in an end-to-end manner from raw sequences. To process data in an unstructured form, we employ graph neural networks (GNNs), which have recently gained considerable attention and have been successfully applied to several bioinformatics problems related to graph representation learning, including the prediction of protein-protein interaction, prediction of drug response, and prediction of protein structure [30]. At the same time, GNNs have not been used in miRNA–mRNA target prediction, and therefore, to the best of our knowledge, we document the first use of graph representation of the duplex and GNNs as classifiers in this domain. To find a suitable GNN architecture, in this study, we compare three popular node embedding methods: Graph Convolutional Networks, GraphSAGE, and Graph Attention Networks.

We denote the proposed framework as GraphTar to emphasize the use of spatial, graph representation of the duplex. As a final contribution of this work, we validate the method against state of the art by fully reimplementing and reproducing miRAW, DeepMirTar, and miTAR experiments to compare the predictive performance. As mentioned earlier, we believe that expert-based methods have significant limitations due to inaccurate prior assumptions and substantial data preprocessing overhead. Therefore, in this study, we will concentrate on applying GraphTar, along with competing methods, to raw sequences.

## Methods

### Dataset

We utilized the meticulously curated dataset collection from the miTAR study in our experiments. Gu et al. employed experimentally validated pairs from the miRAW and DeepMirTar studies. Data for the miRAW study was originally extracted from Diana TarBase [11] and MirTarBase [31]. Additionally, target site sequences were cross-referenced with PAR-CLIP [32], CLASH [33], and TargetScanHuman 7.1 [34]. Data used in DeepMirTar study was collected from the mirMark [35] and CLASH [33] datasets. To apply the most recent knowledge, Gu et al. filtered out the samples containing miRNAs not present in the 22nd release of the miRBase [36]. From the miTAR dataset, we derived four distinct datasets: two sets designated for training, validation, and testing (referred to as miRAW and DeepMirTar), and two additional independent test sets (referred to as miRAWIn and DeepMirTarIn). Furthermore, the miTAR authors compiled a consolidated dataset named MirTarRAW, composed of 33% of data from the miRAW set and 90% of data from the DeepMirTar set. This resulted in a unified dataset containing an equal number of samples from the miRAW and DeepMirTar sets. Each sample within the dataset comprises a pair of miRNA and mRNA sequences, wherein nucleotides are labeled using nucleic acid notation (characters from the set {A, C, G, T, U}), along with a corresponding binary label. A label of 1 indicates a positive interaction, i.e., the miRNA targets the respective mRNA, while a label of 0 signifies a negative interaction. The comprehensive statistics of each dataset used in our study is presented in Table 1. It is noteworthy that we decided to evaluate the methods only using miRAW, DeepMirTar, and MirTarRAW datasets owing to a low number of samples in the independent datasets.

**Table 1:** The number of positive and negative of interactions for each dataset

Dataset	Positive pairs	Negative pairs
miRAW	31,659	30,993
DeepMirTar	3908	3850
MirTarRAW	13,859	13,860

### Sequence encoding

#### Reproduced methods

To classify duplex sequences using the reproduced target prediction methods, we first encoded them using one-hot encoding. This resulted in each nucleotide being represented numerically with a sparse, one-hot vector. Since the mRNA sequences contain Thymine and miRNA sequences contain Uracil, we unified the representation by using the same sparse vector to represent both nucleotides. To ensure a consistent input size, a padding nucleotide ’N’ was added, which has its own sparse representation. To account for this additional padding, one-hot vectors were set to a dimensionality of 5. To evaluate the reproduced methods in our experiments, we padded the miRNA sequences with this nucleotide to the length of the longest miRNA in the respective dataset, and repeated the same process for the mRNA sequences.

#### The proposed method

To prepare encoded inputs for the proposed method, we applied a word2vec-based encoding technique. Word2vec [29] emerged in the natural language processing domain in 2013 as a way to create a vector representation of words. In one of its variants, the Continuous Bag of Words (CBOW) neural network model is trained to predict a selected word based on the context, which is a set of surrounding words in the given sentence. The model can be trained on a set of sentences, each consisting of words from a certain corpus. After training, the hidden layer weights of the model can be considered a lookup table, where each row of the weight matrix at the respective position of the word contains its latent vector representation, learned by the model. The power of this method comes from the fact that words with similar meanings are usually characteristic of similar contexts. On this basis, word2vec can capture functional relationships in language and place synonyms close to each other in the latent space, whereas words with a likely occurrence in different contexts are placed far apart. This trait can also be used in the analysis of biological sequences, as demonstrated by Asgari and Mofrad [37].

To use this method with duplex sequences, we first extracted all miRNA and mRNA sequences from the training dataset into distinct sets. Next, we divided each sequence into 3 nucleotide words in both groups. This way, sequences could be regarded as sentences consisting of words. If the sequences are not divisible by 3, we left 1 and 2 nucleotide remainders as words. Finally, we trained a distinct CBOW model on each set of sentences, one on miRNA sequences, and one on mRNA sequences. As a result, for each word in the dataset, we obtained a dense vector representation by inferring it through a corresponding model. We set the dimensionality of the resulting vectors to 16.

With the trained models in place, the input to our method could be prepared by first splitting the sequence into words and then inferring words through a corresponding CBOW model. Finally, we stacked the resulting dense vectors. With this encoding procedure, one dataset sample consisted of two stacks of dense vectors, one for each sequence in the duplex pair. Note that we conducted a separate experiment for each dataset (DeepMirTar, miRAW, and MirTarRAW), and therefore trained a total of six word2vec models. Moreover, we trained these models only on the respective training datasets to prevent any knowledge from leaking from the test samples.

### Graph classification with GNNs

#### Classifier overview

**Fig. 1:**
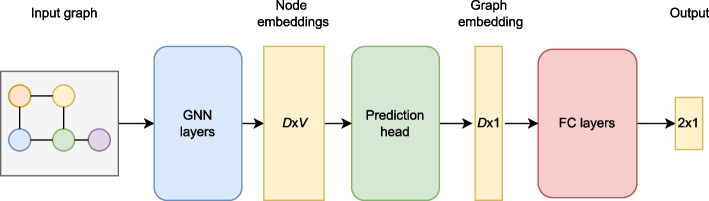
High-level model overview: the input graph is created from the word2vec-encoded sequences. GNN layers process the graph and yield accurate node embeddings, resulting in a graph representation vector. The prediction head operator aggregates the embeddings, and finally, a set of fully connected layers classify the vector. Note that we provide dimension information: *V* denotes the number of nodes in the input graph, while *D* stands for the node embedding dimension, which is equal to the number of output channels of the last GNN layer

The high-level overview of the GraphTar method is presented in Fig. 1. Firstly, the word2vec encoded sequences of the duplex are used to create an input graph in the input graph preparation procedure. The resulting graph representation is then passed through a set of GNN layers, which results in latent graph node space embeddings. Next, the node embeddings are aggregated using a prediction head operator, to form a graph embedding. Finally, the graph embedding is passed through a set of fully connected layers, resulting in a prediction vector.

#### Input graph preparation

**Fig. 2:**
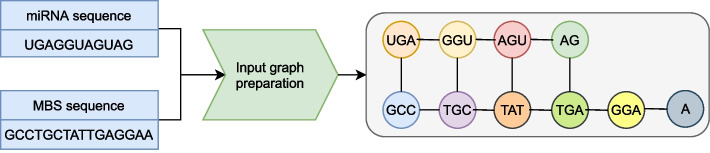
In the process of preparing the input graph, we initially segment the sequences of both the mRNA and miRNA’s Minimal Binding Site (MBS) into triplets. Subsequently, the sequences are aligned based on their starting triplets. Each triplet constitutes an individual graph node, and the nearest nodes are linked by bidirectional edges to construct an undirected graph. This graph functions as the input for GraphTar. It’s important to mention that prior to the graph preparation procedure, the sequence’s triplets were transformed into dense vectors using CBOW models

To create a graph representation of the miRNA–mRNA duplex under consideration, we treat the word2vec encoded words as nodes of the graph. To construct an undirected graph, we align the sequences by the starting word and connect the closest words within and between the sequences with a bidirectional edge. This process is illustrated in Fig. 2.

#### GNN layers

The proposed classifier is composed of two parts. The first part consists of a set of GNN layers, which can process the miRNA–mRNA duplex graph created in the input graph preparation procedure. During training, the layers learn to extract accurate node embeddings, which can be used for various machine learning tasks. Many GNN layer variants have been proposed in the literature. For this study, we selected three established approaches for node embedding extraction: Graph Convolutional Networks (GCNs), the GraphSAGE inductive framework, and Graph Attention Networks (GATs) for evaluation.

GCNs [38] were originally introduced by Kipf and Welling for semi-supervised node classification. The approach is motivated by an approximation of spectral graph convolutions [39]. The layer-wise propagation rule introduced in their work can operate directly on graphs and was able to solve semi-supervised node classification problems on citation networks with state-of-the-art results. For an input graph with *V* nodes with features *X*, the layer-wise propagation rule proposed in their work is:

$$\begin{aligned} H^{(l+1)}=\sigma ({\tilde{D}}^{-\frac{1}{2}}{\tilde{A}}{\tilde{D}}^{-\frac{1}{2}}H^{(l)}W^{(l)}) \end{aligned}$$

where ${\tilde{D}}$ is a graph degree matrix, ${\tilde{A}}$ is the adjacency matrix, $H^{(l)}\in {\textbf{R}}^{V\times {D}}$ is a matrix of node features at layer *l*; $H^{(0)} = X$. $W^{(l)}$ is a trainable weight matrix. This method can be generalized to unseen graphs, as all trainable weights at each layer can be shared between the nodes. The weights can be then used during inference on unseen nodes.

Hamilton, Ying, and Leskovec extended the idea behind GCNs by proposing a general, inductive framework for node embedding—GraphSAGE [40]. In GraphSAGE, the forward propagation algorithm is also layer-wise and makes use of two operators: aggregation (*AGG*) and concatenation (*CONCAT*). For graph with *V* nodes, the steps leading to the computation of the node *v* feature *h* at layer *l* (denoted as $h_v^l$) are as follows:

$$\begin{aligned} h_{{\mathcal {N}}(v)}^l= & {} AGG_l(\{h_u^{l-1}, \forall {u} \in {\mathcal {N}}(v)\}, \\ h_v^l= & {} \sigma \left( W^l \cdot CONCAT(h_v^{l-1}, h^l_{{\mathcal {N}}(v)})\right) \\ h_v^l= & {} h_v^l/||h_v^l||_2 \end{aligned}$$

Note, that again, if we denote the original feature vector of node *v* as $x_v$, then $h_v^0=x_v$ In the first step, the node neighborhood ${\mathcal {N}}(v)$ is passed to the *AGG* operator, which yields a representation of the neighborhood $h^l_{{\mathcal {N}}(v)}$. The aggregation operator can be simply a computation of the element-wise mean of the neighbor feature vectors at the previous layer, but the framework is flexible and more complicated functions can be used. In the original article, authors used also an LSTM aggregator and a pooling aggregator, consisting of a fully connected neural network and an element-wise max-pooling operation. In the second step of the forward pass, the node *v* latest representation $h_v^{l-1}$ is concatenated with its neighborhood representation. The result is then multiplied with a weight matrix at the respective layer $W^l$, shared between all nodes. This gives the framework an inductive characteristic - it is able to be generalized to unseen nodes. Finally, a non-linearity is applied and the vector is normalized to unit length. It is worth noting, that aggregation of the node neighborhood may be expensive for large graphs. to solve this issue, the authors propose to sample a fixed-size neighborhood, instead of taking into account all neighbors.

Graph Attention Networks [41] were proposed by Veličković et al, as an alternative for spectral (e.g. GCNs) and non-spectral (e.g. GraphSAGE) node embedding approaches. GATs use a self-attention mechanism which allows the network to choose important nodes in the node’s neighborhood. For this purpose, at each layer, first the attention coefficients are calculated. Using neighborhood notation from the GraphSAGE description, for node *v*, the attention coefficient $e_{vu}$ is calculated as follows:

$$\begin{aligned} e_{vu} = a \left( W_k h_u^{k-1}, W_k h_v^{k-1} \right) , \forall u, u \in {\mathcal {N}}(v) \end{aligned}$$

Where $w_k$ is a weight matrix applied to every node and shared within a layer *k*, and $h_v^{k-1}$ is a feature vector of node *v* at layer $k-1$. The attention mechanism *a* is a fully connected neural network with LeakyReLU nonlinearity. After computing the attention coefficients, they are normalized using the softmax function, so that the resulting neighbor importances sum to 1:

$$\begin{aligned} \alpha _{vu} = \frac{exp(e_{vu})}{\sum _{n\in {\mathcal {N}}(v)}exp(e_{vn})} \end{aligned}$$

Finally, the normalized attention coefficients can be used to to calculate node feature vector $h_v^k$, after applying a non-linearity:

$$\begin{aligned} h_v^k=\sigma \left( \sum _{u\in {\mathcal {N}}(v)} \alpha _{vu}W_kh_u^{k-1}\right) \end{aligned}$$

In our experiments, we evaluate the use of the aforementioned node embedding approaches, to compare their performance. Regardless of the approach used for a graph with *V* nodes, GNN layers yield a $D \times V$ embedding matrix, where *D* is the dimensionality of the node feature vectors. *D* is one of the hyperparameters that has to be tuned to obtain the best performance.

#### Prediction head

Node features extracted by the GNN layers contain important information about the nodes and can be used for various tasks, including node, edge, and graph classification. The prediction head operator transforms these embeddings into a suitable representation for the chosen task. In this study, we aim to solve a graph classification problem, and thus, we need to acquire a graph representation based on the node embeddings that can be classified by a set of fully connected layers. To achieve this goal, we apply a pooling operator that aggregates the node information into a graph feature vector with *D* features, which is equal to the dimensionality of the node feature vectors. We consider three classic pooling operators, established in the literature [38, 40]: global add pooling (*ADD*), global mean pooling (*MEAN*), and global max pooling (*MAX*), and choose the best one for each embedding approach during the hyperparameter tuning process. These operators apply element-wise sum, averaging, or maximum operations to the node feature vectors, yielding an aggregated graph representation. The operators preserve different aspects of the graph. For instance, the global add pooling provides distinct graph embeddings for graphs of varying sizes, as a greater number of nodes will generally provide greater element-wise sum values. On the other hand, global mean pooling can provide similar graph embeddings, even if the considered graphs vary greatly in size—when averaging, similar element-wise values can be obtained for both large and small graphs.

#### Fully connected layers

The vector with graph embedding is processed by a set of fully connected layers, with ReLU activations. As a regularization mechanism, we use Dropout [42] after every but the last fully connected layer. The final layer returns two values and is normalized using the softmax function. This yields two probability values, indicating, whether the considered graph is an instance of a positive, or negative interaction.

### Evaluation metrics

To evaluate the performance of the reproduced methods and the GNN classifiers proposed in this work, we report the following metrics: Balanced accuracy (BACC), Precision, Recall. These metrics are calculated as follows:

$$\begin{aligned} BACC= & {} \frac{\frac{TP}{TP + FN} + \frac{TN}{TN + FP}}{2}, \\ Precision= & {} \frac{TP}{TP + FP}, \\ Recall= & {} \frac{TP}{TP + FN}, \end{aligned}$$

where TP, TN, FP, and FN are true positives, true negatives, false positives, and false negatives, respectively. Balanced accuracy is a metric that indicates the percentage of correctly classified labels. Precision indicates how many miRNA:mRNA pairs classified as instances of positive interaction are actually positive. Recall is a metric that shows the percentage of positive pairs in the dataset that were classified as positive.

### Implementation and availability

The data preparation and preprocessing were implemented in Python 3.8.10 [43] using the Pandas package 1.3.0 [44]. For the reproduced methods, we used the PyTorch machine learning framework 1.9.0 [45]. To implement graph neural networks, we used the PyTorch Geometric extension 1.7.2 [46]. Word2vec embeddings were prepared using utilities provided by the Gensim package 4.0.1 [47]. Training was implemented using PyTorch Lightning 1.5.10 [48]. The datasets, trained models and results data, together with the code and reproduction steps, are available in the project repository [49].

## Results

### Experimental setup

To compare the proposed approach with the current state of the art, we have reimplemented miRAW [26], DeepMirTar [24] and miTAR [27] according to descriptions from the respective articles and evaluated it on data described in Table 1 together with the proposed GraphTar approach. Note, that for DeepMirTar we used only a part of the proposed architecture—we employed only raw seuqences as input to the model, whereas in the original study expert-based features were also used in addition. Within the GraphTar framework, we set to train a separate model for each of the node embedding methods considered ($E_{GNN} \in [GCN, GAT, GraphSAGE]$), with the resulting models denoted as *GraphTarGCN*, *GraphTarSAGE* and *GraphTarGAT* respectively. For each compared method, we trained three separate models on miRAW, DeepMirTar, and MirTarRAW datasets, split using training:validation:test ratio of 0.7:0.15:0.15. We repeated this step for 30 data splits, which resulted in 90 models overall for each method. We train all models for 1000 epochs using Adam optimizer [50], starting with learning rate $lr=0.001$ and reducing it on plateau. If the model did not improve for 100 epochs, we performed early stopping.

### Hyperparameter tunning

To obtain the best performance, for all GNN layer types, prior to the experiments, we performed a hyperparameters search on the MirTarRAW dataset on one data split using the grid search methodology. The selection was based on the balanced accuracy score on the validation set. We searched for the best GNN layer embedding size $D_{GNN}\in [16, 32, 64, 128, 256, 512]$, as well as the optimal number of graph layers $L_{GNN} \in [1, 2,\ldots ,10]$. Similarly, we attempted to find the best prediction head operator $P_h \in [ADD, MEAN, MAX]$ for each of the node embedding methods. Using the same parameter ranges, we also tuned the dimensionality of fully connected layers and their number ($D_{FC}$ and $L_{FC}$ respectively), as well as dropout rate $R_{D} \in {0.2, 0.3, 0.4, 0.5, 0.6}$. The resulting parameters used in GraphTar experiments are provided in Table 2. For all models, including the reproduced methods, we also found the optimal batch size $B_s\in [16,32,64,128,256,512]$ for each training dataset (Table 3).

**Table 2:** Optimal set of parameters for each node embedding method considered in the study

$E_{GNN}$	$L_{GNN}$	$D_{GNN}$	$L_{FC}$	$D_{FC}$	$P_h$	$R_{D}$
GCN	3	128	2	512	MAX	0.4
GAT	5	256	2	128	ADD	0.4
GraphSAGE	5	256	3	256	ADD	0.4

**Table 3:** Batch sizes used in each model for DeepMirTar, miRAW and MirTarRaw datasets

Model	DeepMirTar	miRAW	MirTarRAW
miRAW	128	64	128
DeepMirTar	512	512	256
miTAR	128	128	128
GraphTarGCN	128	64	64
GraphTarSAGE	128	32	256
GraphTarGAT	128	512	512

**Table 4:** DeepMirTar dataset results

Model name	Balanced ACC	Precision	Recall
DeepMirTar	0.815 (0.811–0.819)	0.817 (0.812–0.821)	0.815 (0.811–0.819)
miRAW	0.902 (0.898–0.907)	0.902 (0.897–0.908)	0.902 (0.898–0.907)
**miTAR**	**0.927 (0.923–0.932)**	**0.928 (0.924–0.932)**	**0.927 (0.923–0.932)**
GraphTarGCN	0.904 (0.899–0.909)	0.905 (0.899–0.91)	0.905 (0.899–0.91)
GraphTarGAT	0.922 (0.917–0.927)	0.923 (0.917–0.928)	0.922 (0.917–0.927)
GraphTarSAGE	0.915 (0.909–0.922)	0.916 (0.909–0.923)	0.915 (0.909–0.922)

Metric scores are shown with corresponding 0.95 CIs

**Table 5:** MiRAW dataset results

Model name	Balanced ACC	Precision	Recall
DeepMirTar	0.875 (0.874–0.876)	0.877 (0.875–0.878)	0.875 (0.874–0.876)
miRAW	0.938 (0.937–0.939)	0.939 (0.938–0.94)	0.938 (0.937–0.939)
miTAR	0.939 (0.938–0.941)	0.94 (0.939–0.942)	0.939 (0.938–0.941)
GraphTarGCN	0.915 (0.934–0.937)	0.916 (0.935–0.938)	0.915 (0.934–0.937)
**GraphTarGAT**	**0.948 (0.947–0.949)**	**0.949 (0.947–0.95)**	**0.948 (0.947–0.949)**
GraphTarSAGE	0.94 (0.939–0.941)	0.941 (0.939–0.942)	0.94 (0.939–0.941)

Metric scores are shown with corresponding 0.95 CIs

**Table 6:** MirTarRaw dataset results

Model name	Balanced ACC	Precision	Recall
DeepMirTar	0.827 (0.825–0.828)	0.826 (0.824–0.828)	0.827 (0.825–0.828)
**miRAW**	**0.928 (0.927–0.929)**	**0.928 (0.927–0.929)**	**0.928 (0.927–0.93)**
miTAR	0.91 (0.907–0.913)	0.91 (0.907–0.914)	0.91 (0.907–0.913)
GraphTarGCN	0.915 (0.913–0.918)	0.915 (0.913–0.918)	0.915 (0.913–0.918)
GraphTarGAT	0.921 (0.918–0.922)	0.921 (0.918–0.922)	0.921 (0.918–0.922)
GraphTarSAGE	0.914 0.912–0.916)	0.914 (0.912–0.916)	0.914 (0.912–0.916)

Metric scores are shown with corresponding 0.95 CIs

### Performance versus the state of the art

In this section, we present our findings on the performance comparison between the GraphTar models and state-of-the-art methods. Our results, which are summarized in Tables 4, 5, and 6, reveal that there is no method that performs better than others on all datasets.

We observed that the GraphTar models, along with miRAW and miTAR, consistently outperformed the adaptation of DeepMirTar in terms of balanced accuracy score, with the differences ranging from 0.04 to 0.083, depending on the dataset. On the DeepMirTar dataset, it was miTAR model that achieved the best performance with a balanced accuracy score of 0.927, followed by GraphTarGAT (0.922) and GraphTarSAGE (0.915). GraphTarGCN and miRAW achieved considerably lower scores of 0.904 and 0.902, respectively, while DeepMirTar performed the worst with an accuracy score of 0.815.

On the miRAW dataset, GraphTarGAT emerged as the top-performing method with a balanced accuracy score of 0.948, followed closely by GraphTarSAGE, miTAR, and miRAW with scores of 0.94, 0.939 and 0.938, respectively. GraphTarGCN and DeepMirTar exhibited inferior performance with balanced accuracy scores of 0.915 and 0.875.

**Fig. 3:**
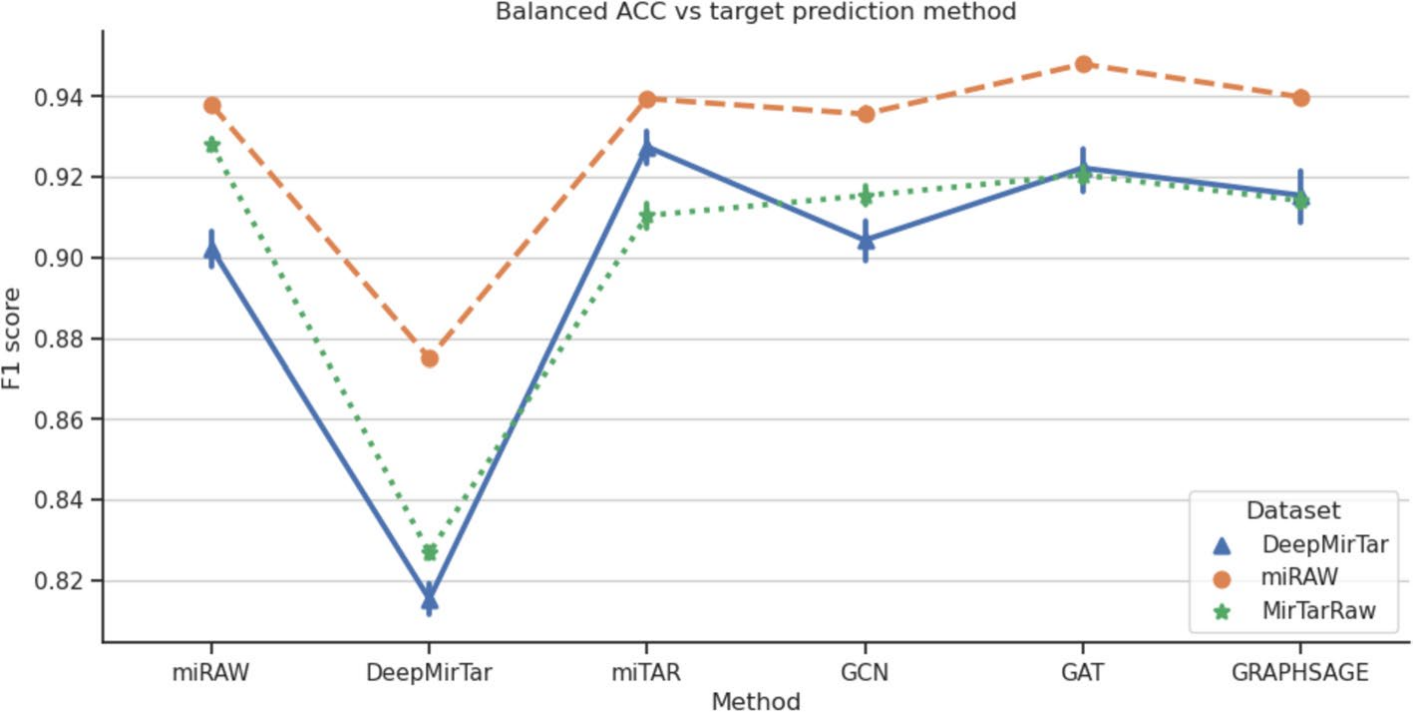
A plot visualizing models performance on test datasets. Among these datasets, miTAR emerged as the top-performing method for the DeepMirTar dataset. On the miRAW dataset, the most effective approach was GraphTarGAT (abbreviated as GAT), while for the combined MirTarRaw dataset, the highest metric scores were achieved by miRAW. The abbreviations GCN and GRAPHSAGE correspond to GraphTarGCN and GraphTarSAGE, respectively

Finally, on the MirTarRaw dataset, miRAW achieved the highest balanced accuracy score of 0.928, while GraphTarGAT came in second with a score of 0.921. GraphTarGCN, GraphTarSAGE, and miTAR obtained balanced accuracy scores of 0.915, 0.914, and 0.91, respectively. In contrast, DeepMirTar performed worst with a balanced accuracy score of 0.827. An illustration of the results is shown on Fig. 3.

### Ablation experiments

**Fig. 4:**
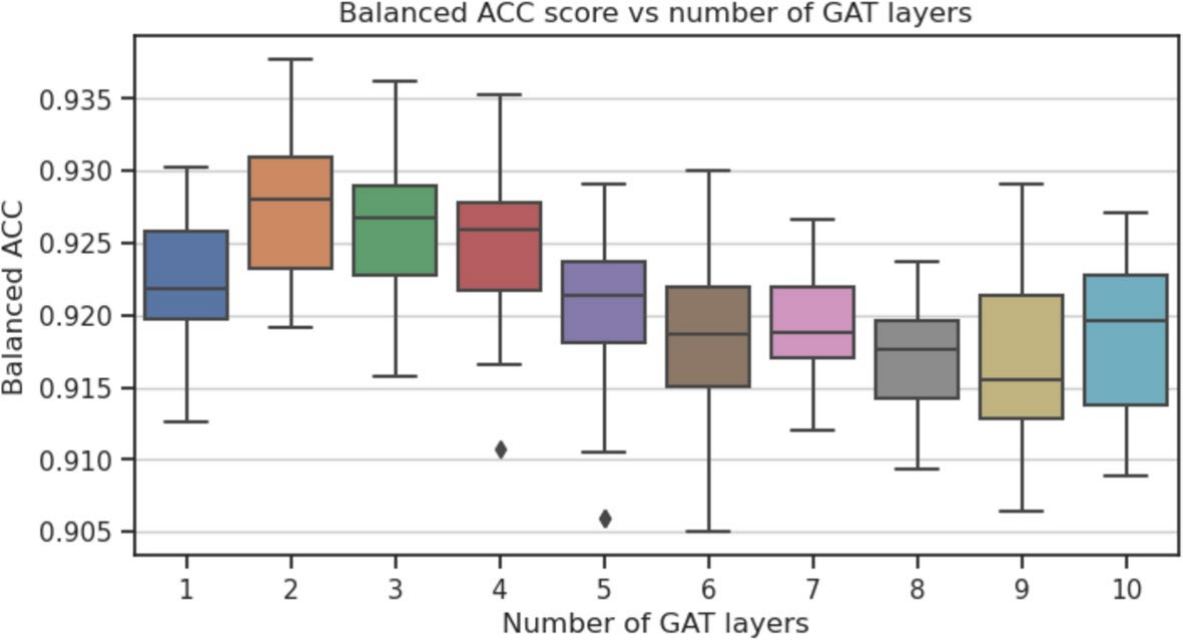
The impact of variable number of GAT layers on balanced accuracy score on the MirTarRaw dataset

**Fig. 5:**
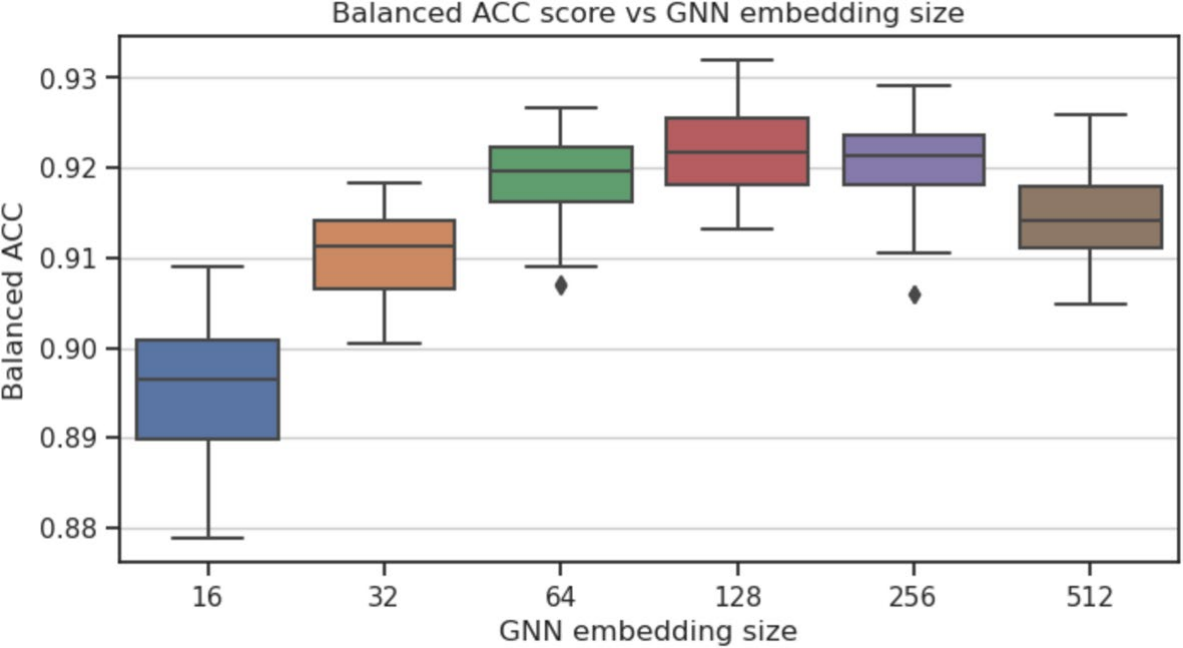
The impact of variable GNN embedding size on balanced accuracy score on the MirTarRaw dataset

**Fig. 6:**
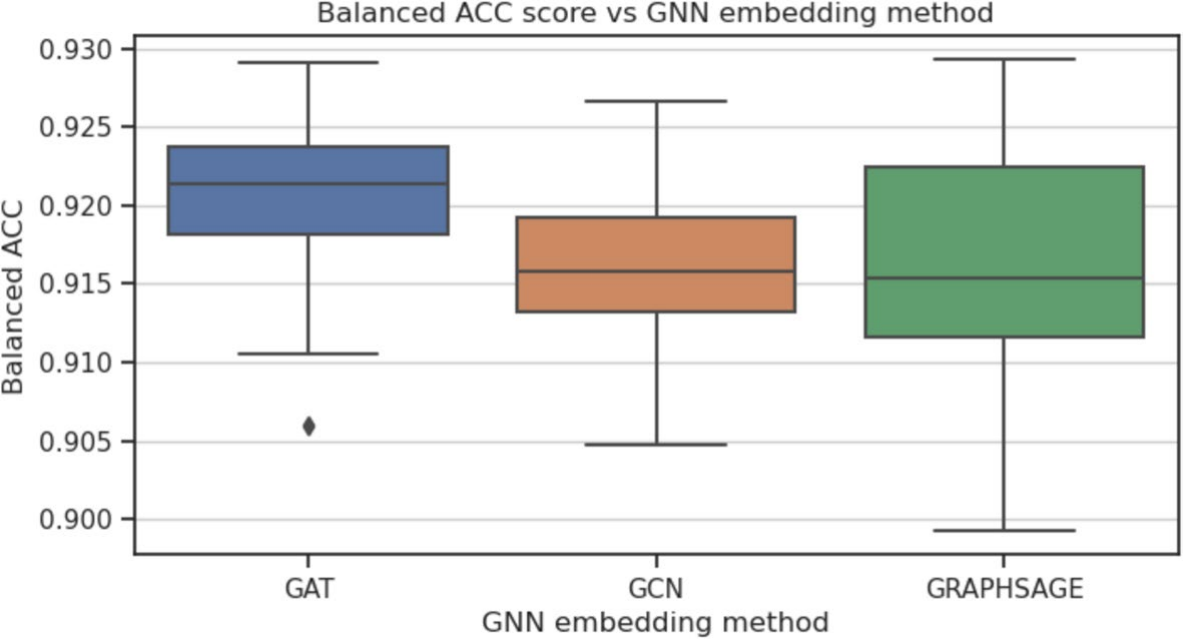
The impact of GNN embedding method on balanced accuracy score on the MirTarRaw dataset

**Fig. 7:**
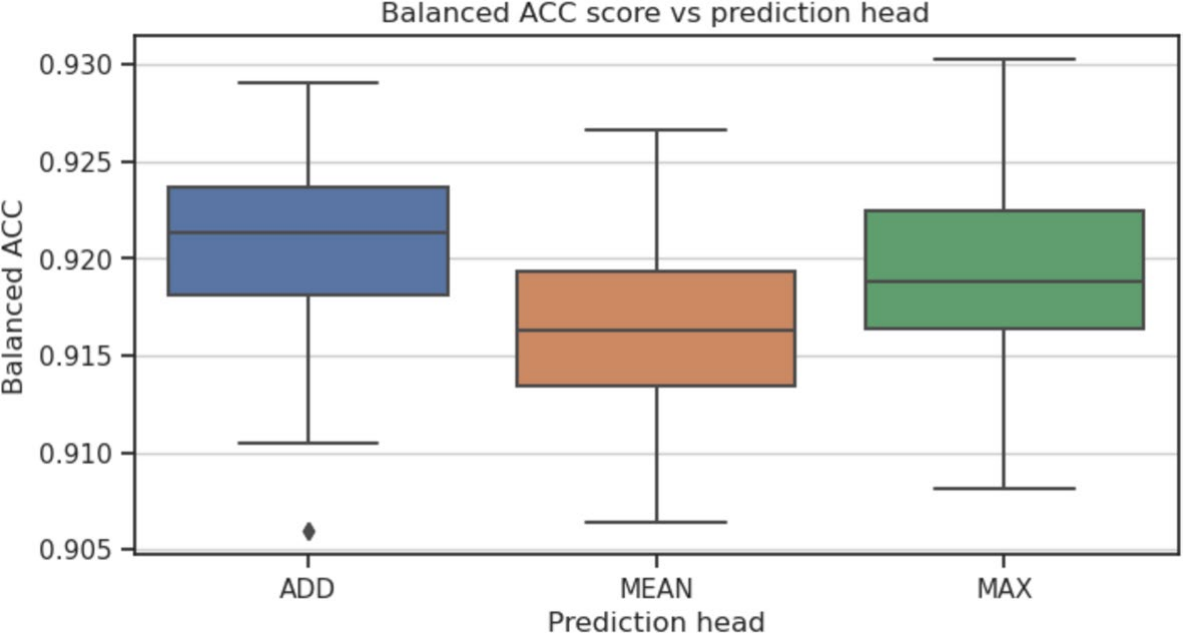
The impact of prediction head type on balanced accuracy score on the MirTarRaw dataset

To investigate which parts of the GraphTar architecture have the greatest impact on predictive performance, we conducted ablation experiments. In these experiments, we assessed the effects of four aspects of the graph encoder architecture: the number of graph embedding layers, graph layer embedding size, graph embedding method, and the prediction head. The baseline for our experiments was GraphTarGAT with the following parameters: $E_{GNN}=5$, $L_{GNN}=5$, $D_{GNN}=256$, $L_{FC}=2$, $D_{FC}=128$, $P_h=ADD$, $R_D=0.4$. We carried out the experiments using the MirTarRaw dataset, given its substantial sample size. For each experiment, all utilized model configurations were trained and evaluated across 30 data splits, following the methodology employed in the preceding experiments. The parameters we considered included: $D_{GNN}\in [16, 32, 64, 128, 256, 512]$, $L_{GNN} \in [1, 2,\ldots ,10]$, $P_h \in [ADD, MEAN, MAX]$ and $E_{GNN} \in [GCN, GAT, GraphSAGE]$ As a result of ablation study, we observed that the optimal number of GNN layers was equal to 2. Having more than 2 GAT layers (balanced accuracy equal to 0.927) resulted in a decreased metric scores (Fig. 4), with the lowest score equal to 0.916 for $L_{GNN}=9$. As for the graph layer embedding size, the results show, that the optimal value was in the middle of our search space - equal to 128 (balanced accuracy equal to 0.92), as seen on Fig. 5). In this experiment, the lowest value recorded was obtained with $D_{GNN}=16$ and equal to 0.895. Out of three GNN embedding methods considered (Fig. 6), the best results were obtained with GAT (0.92) and the worst for GCN (0.916). Finally, the best performing prediction head was global add pooling (0.92 accuracy score), whereas the worst performing global mean pooling obtained 0.916 accuracy score (Fig. 7).

## Discussion

### Performance versus the state of the art

In this study, we compared the performance of several machine learning models, including the proposed GraphTar method, on three datasets: DeepMirTar, miRAW, and MirTarRaw. Our results indicate, that different methods obtained the best performance on different datasets, with the miRAW model yielding the best results on the MirTarRaw dataset, while miTAR was the best on the DeepMirTar dataset. Interestingly, our findings did not support the results of the miTAR article [27], which indicated that this method was clearly superior to miRAW.

We observed that all methods exhibited very similar performance, except for the DeepMirTar method, which clearly demonstrated less proficiency in target prediction. This could be a result of an inferior architecture being employed. However, it’s important to note that our study focused on methods operating with raw sequence inputs. Consequently, we modified the architecture to exclude expert-based features from the input, as opposed to the original study. This modification likely had an impact on the performance of DeepMirTar models.

We discovered that the proposed GraphTar method matched, at the very least, the performance of miRAW and miTAR. It achieved the best performance on the miRAW dataset, the second-best on the MirTarRaw dataset, and performed at par with miTAR on the DeepMirTar dataset. This implies that the novel graph representation can effectively describe the spatial structure of a miRNA–mRNA duplex. It also indicates that graph neural networks can be as effective in target prediction as architectures built on autoencoders and recurrent layers. However, we couldn’t establish a clear, dataset-independent advantage of the GNN architecture over state-of-the-art methods. Further development, including the exploration of novel node embedding methods, meticulous parameter tuning, and experimentation with various GNN architectures, holds significant promise for the future.

Additionally, the results unveiled that among the considered node embedding methods, GraphTar, based on Graph Attention Networks, clearly outperformed the others. The attention mechanism proved to be the most effective in learning node embeddings. Following closely was the GraphTarSAGE method, with GraphTarGCN only posing a challenge to GraphTarSAGE on the MirTarRaw dataset.

Interestingly, we discovered that graph neural networks could only achieve performance comparable to miTAR and miRAW when utilizing word2vec embeddings in the graph representation. Our initial experiments demonstrated that GNN-based models, when their input was encoded with one-hot encoding, failed to surpass a balanced accuracy score of approximately 0.85 across all datasets. This indicates that word2vec-based encoding dramatically improves the performance of GNNs in this context, compared to other encoding techniques, like one-hot encoding. It also suggests that while GNNs excel at generating accurate graph embeddings for duplex structures, they struggle to precisely capture the features of sequence elements at the nucleotide level. Further investigation should center on understanding the specific enhancements introduced by the word2vec embedding method and the limitations of GNNs in embedding biological sequences. Moreover, there exists a necessity to thoroughly investigate and interpret the acquired representations within GNN-based models to unveil the inherent characteristics of the miRNA–mRNA duplex. These insights could provide valuable comprehension of the miRNA binding mechanism and offer a deeper understanding of the biological processes underpinning target prediction. Clearly, explainability is important for researchers, which is why easy-to-interpret algorithms are popular in computational biology. An example could be Ordinary Differential Equations-based (ODE-based) modeling methods employed in systems biology, such as in studies on protein signaling networks [51, 52]. From our perspective, ensuring the interpretability of GNN models represents a crucial avenue of research for any interaction prediction study employing this category of deep learning architectures. Although in recent years, GNN-based prediction methods have become more popular in other fields of computational biology, such as metabolite-disease associations (Sun et al., [53]), long non-coding RNA (Wang et al., [54]), and protein-protein interactions (Shen et al., [55]), little research was focused on actually investigating the intrinsic mechanisms behind their predictive performance. One reason for this could be that the methodology of such investigations is not widely understood. To address this, providing a set of good practices and methods for GNN model interpretability in the context of interaction prediction could give the momentum necessary to comprehend and utilize the knowledge conveyed within the prediction models.

Once understood, this information could further enable the identification of novel drug targets, genetic markers, and disease associations. Another angle that supports the importance of interpretability is the fact that it is commonly referenced as a challenge that prevents the use of deep learning methods in healthcare (Miotto et al., [56]). In the current state of interaction prediction research, where the models cannot be used in practice (e.g., applied to real patients in therapies), it is much more interesting to know *why* various interactions occur than *if* they occur. The classification task itself is merely a way to guide the models to learn the right task to solve, but for the field, it is not as important as deepening the understanding of the interaction intrinsics. Once we deepen our understanding of intrinsics, we can design better data preprocessing methods and models, and crucially validate them using this knowledge. At this point, when we are able to understand the models, we can convince medical professionals to use them in practice, and then the *if* question will really become relevant.

### Ablation experiments

One methodology that could be of great help in revealing crucial components of the GNN architecture involves conducting ablation experiments, similar to the ones documented in the results section. Through these experiments, we were able to observe how different aspects of the graph encoder (such as the number of layers, embedding size, prediction head, and layer type) impact predictive performance. We deduced that while all these aspects influence the results, the most significant ones were the embedding size and the number of GNN layers.

Interestingly, ablation experiments focused on these aspects revealed which parameters found by our hyperparameter tuning procedure could be still potentially further improved and which seem to have the best values. They indicate that we might have achieved superior results by using 2 GAT layers instead of 5 and a graph embedding size of 128 instead of 256 in the evaluated GraphTarGAT architecture in the “Performance vs the state of the art” experiments. To improve the process, it would require performing the hyperparameter tuning procedure on multiple data splits, instead of one. However, this would significantly increase the amount of computations required in this step.

It seems that a 128-dimensional embedding adequately encapsulates essential information about the nodes in the input graph for the miRNA–mRNA duplex. The optimal choice of 2 layers suggests that the graph neural network needs to propagate information from neighbors at most two edges away from a node to obtain the most informative embedding. This suggests that nucleotide interactions primarily occur in close proximity and are not widely distributed across the duplex.

For the selection of prediction heads, we found that the best performance in the ablation experiment was achieved with the *ADD* operator, followed by *MAX* and *MEAN*. Meanwhile, the average balanced accuracy score gap between the best and worst performing operators is not substantial, equaling 0.05. It remains unclear why the *ADD* operator produced the best results and what the underlying mechanisms for this performance are. As previously mentioned, an investigation into model interpretability could potentially unveil and elucidate the impact of various prediction heads on the model’s predictions. Given that pooling operators are a crucial component of GNN classifiers, this constitutes an important direction for future research.

In summary of these experiments, when fine-tuning GNN architectures, we recommend commencing the process by adjusting the number of graph embedding layers, followed by tuning the embedding size and prediction head. Only subsequently should you proceed to search for the most optimal graph embedding layer type.

### Dataset quality

While the miRAW and DeepMirTar datasets have provided a valuable benchmark for our study, it is crucial to expand the current datasets to propel target prediction methods towards real-world applications. The biggest limitation of DL methods is related to data quality, therefore in numerous domains of computational biology, DL-based interaction prediction algorithms face constraints. Based on our expertise in this domain, we can delineate data quality limitations as follows:

- Data availability: large interaction datasets are not common and widely available.
- Data balance: the ratio of positive and negative samples is highly imbalanced (e.g. in miRNA–mRNA interaction prediction, there is a complete lack of experimentally validated negative samples, whereas in metabolite-disease association prediction, the ratio of positive to negative samples can be 1–100, as outlined by Sun et al. in [53]). Data imbalance makes models biased and difficult to train and evaluate.
- Benchmarking: there is no established, official benchmark to evaluate miRNA–mRNA interaction prediction methods, which makes comparing them difficult and unreliable.
- Data heterogeneity: interaction studies cover a small part of the tissue and disease search space. Conducting a wider search and expanding knowledge about the impact of interaction phenomena can uncover their influence on diseases that so far were out of the spotlight in this research field.

Considering the aforementioned deficiencies, forthcoming studies should prioritize the acquisition of more diverse, well-balanced, and extensive datasets. This effort will establish a robust foundation for the trustworthy evaluation of data-driven prediction algorithms like GraphTar. An alternative could involve turning to more data-efficient approaches, such as the aforementioned ODE-based modeling methods [51, 52]. Employing methods that can work with a modest dataset could prove essential in advancing the state of the art, regardless of the challenges in dataset collection. Nevertheless, the presence of standardized evaluation datasets and methodologies holds paramount importance for employing computational techniques as research instruments and for their potential applications in healthcare contexts.

## Conclusions

In this study, we introduced an innovative approach to miRNA target prediction named GraphTar. We framed target prediction as a graph classification problem and put forth a novel graph representation for the miRNA–mRNA duplex. For encoding the nucleotide triplets within each sequence, we harnessed the word2vec method, which had not been previously employed in target prediction. The resulting graph, composed of encoded triplets, was subjected to classification using a graph neural network. Through a comprehensive comparison with replicated state-of-the-art methods, we illustrated that GraphTar can match the performance of state-of-the-art classifiers and even surpass them on one of the datasets. To gain valuable insights, we conducted ablation experiments, assessing the influence of graph layer count, their type, as well as embedding size and global pooling method on predictive performance. Building upon our experience, we discussed the most important future study directions, such as exploring the underlying mechanisms of GNN-based interaction prediction methods and developing more accurate and efficient GNN architectures. As outlined in the discussion section, expanding and standarizing the available datasets for target prediction will also be critical to advance the field towards real-world applications.

## Data Availability

The datasets, trained models and results data, together with the code and reproduction steps, are available in the project repository [49].

## Abbreviations

CNNs: Convolutional neural networks
RNNs: Recurrent neural networks
DL: Deep learning
BiRNN: Bi-directional RNN
LSTM: Long short-term memory
PPV: Positive predictive value
TP: True positives
TN: True negatives
FP: False positives
FN: False negatives
GNNs: Graph Neural Networks
GATs: Graph Attention Networks
GCNs: Graph Convolutional Networks
AGO: Argonaute protein
miRISC: MiRNA-induced silencing complex
RNA: Ribonucleic acid
miRNAs: Micro RNAs
mRNAs: Messenger RNAs
SVM: Support Vector Machines
ML: Machine Learning
SdA: Stacked denoising autoencoder
NLP: Natural Language Processing
CBOW: Continuous Bag of Words
ODE: Ordinary Differential Equations
